# Thiamine Deficiency Induced Neurochemical, Neuroanatomical, and Neuropsychological Alterations: A Reappraisal

**DOI:** 10.1155/2013/309143

**Published:** 2013-10-21

**Authors:** Raffaele Nardone, Yvonne Höller, Monica Storti, Monica Christova, Frediano Tezzon, Stefan Golaszewski, Eugen Trinka, Francesco Brigo

**Affiliations:** ^1^Department of Neurology, Paracelsus Medical University, Ignaz Harrer-Strasse 79, A-5020 Salzburg, Austria; ^2^Department of Neurology, Franz Tappeiner Hospital, Via Rossini 5, 39012 Merano, Italy; ^3^Department of Medicine, Franz Tappeiner Hospital, Via Rossini 5, 39012 Merano, Italy; ^4^Department of Physiology, Medical University of Graz, Harrachgasse 21/5, A-8010 Graz, Austria; ^5^Department of Neurological, Neuropsychological, Morphological and Movement Sciences, Section of Clinical Neurology, University of Verona, Piazzale Luduvico Antonio Scuro 10, 37134 Verona, Italy

## Abstract

Nutritional deficiency can cause, mainly in chronic alcoholic subjects, the Wernicke encephalopathy and its chronic neurological sequela, the Wernicke-Korsakoff syndrome (WKS). Long-term chronic ethanol abuse results in hippocampal and cortical cell loss. Thiamine deficiency also alters principally hippocampal- and frontal cortical-dependent neurochemistry; moreover in WKS patients, important pathological damage to the diencephalon can occur. In fact, the amnesic syndrome typical for WKS is mainly due to the damage in the diencephalic-hippocampal circuitry, including thalamic nuclei and mammillary bodies. The loss of cholinergic cells in the basal forebrain region results in decreased cholinergic input to the hippocampus and the cortex and reduced choline acetyltransferase and acetylcholinesterase activities and function, as well as in acetylcholine receptor downregulation within these brain regions. In this narrative review, we will focus on the neurochemical, neuroanatomical, and neuropsychological studies shedding light on the effects of thiamine deficiency in experimental models and in humans.

## 1. Introduction

The acute Wernicke's encephalopathy (WE) and the Wernicke-Korsakoff syndrome (WKS) are principally nutrition deficiency disorders due to brain damage caused by a lack of thiamine (vitamin B1), even if they occur most frequently in alcoholic patients [1, 2]. WKS is often, but not always, the chronic neurological sequela after WE, which results from severe acute deficiency of thiamine.

Chronic alcohol consumption leads to two main long-lasting neurological disorders, both associated with severe impairment of cognitive functions: the alcohol-associated dementia (AAD) and the WKS [3]. It has been estimated that greater than 10% of alcoholic patients have symptoms of either AAD or WKS [4, 5].

Postmortem prevalence rates of WKS are 1-2% in the general population and 12–14% in the alcoholic population [6, 7]. Therefore, despite the clear diagnostic criteria of WE, WKS is diagnosed more commonly in alcoholics at post-mortem than when the patients are alive [8]. 

On the other hand, a clear clinical distinction between AAD and WKS is difficult. Mild-to-moderate thiamine deficiency plays a role in the neurodegeneration observed in chronic alcoholics, and a proteomics-based study has revealed that thiamine metabolism may also be altered in non-WKS alcoholics [9]. However, it is often difficult to determine the dietary status of many alcoholic patients, making WKS an underdiagnosed disorder [7, 10]. Moreover, there is considerable overlap between AAD and WKS with regard to both neuropathology and behavioral symptoms [7, 10, 11]. AAD and WKS are also considered to be distinct disorders with overlapping clinical symptoms [12–14]. In the absence of a “gold standard” for the diagnosis of AAD as distinct entity from WKS, the clinical diagnosis is often difficult [7]. The development of these disorders involves numerous factors. It has been hypothesized a genetic predisposition is critical for the development of WKS or AAD. The key factors are thought to be alterations in thiamine metabolism or susceptibility to alcohol neurotoxicity, respectively.

We aimed in this paper to summarize the salient neurochemical, neuropathological, and neuropsychological findings that have been reported in experimental animals and in humans after thiamine deprivation.

## 2. Neuropsychological Findings

The profile of cognitive impairment observed in AAD patients is more variable than that reported in WKS patients. AAD is characterized by a wide range of cognitive deficit, involving perceptual-motor and visuospatial skills, abstraction, and problem solving, as well as learning and memory processes [4, 15]. 

In WKS patients, the key behavioral features are profound amnesia and the appearance of confabulation [1, 2]. New episodic and declarative learning and recall are impaired. Both anterograde and retrograde amnesia are characteristically present in WKS. The inability to recall events prior to the occurrence of the amnesic state can extend back to 20–30 years [16]; there is generally a temporal gradient such that the patients are better able to recall earlier memories than the more recent ones. 

While the amnesic symptoms typically associated with WKS appear acutely due to synergistic effect of alcoholism and severe malnutrition, visuoperceptual deficits and problem solving develop slowly after many years of alcohol abuse [17].

Nevertheless, WKS patients often show, beyond the amnesia, impairment of other cognitive functions, such as verbal fluency, flexibility, and perseverative responding. In particular, even if cognitive deficits on frontal cortical-dependent tasks can be observed in alcoholics with and without WKS [15, 18, 19], WKS patients are frequently impaired on tests sensitive to functions of dorsolateral prefrontal and orbitofrontal subsystems [18]. 

 Episodic memory is typically severely affected in the WKS, while semantic memory is variably affected, and studies assessing perceptual priming as well as motor performances demonstrated that the components of implicit memory remain largely preserved in the WKS [20]. The impairment in delay eyeblink conditioning, a basic form of implicit memory, in WKS and non-WKS alcoholics [21] seems to be related to cerebellar deterioration that occurs after long-term alcohol consumption.

A deficit involving the initial “encoding” information in WKS has been theorized. Indeed, patients with WKS are able to encode direct and sensory impressions, while the processing of semantic material is difficult to perform [22]. On the contrary, procedural memory is relatively intact. Moreover, an impairment in the physiological processes underlying the consolidation of information in a more permanent form of storage has been postulated [23, 24]. In agreement with this hypothesis, scores on the tests measuring primary or working memory (such as the span test) are characteristically intact in WKS patients [25]. Alternatively, since WKS patients show a disproportionately impaired recall of the spatial or temporal aspects of learned material [26, 27], also a specific deficit in the storage of contextual information should be taken into consideration. On the other hand, WKS patients may show a retrieval deficit and thus be unable to suppress inappropriate responses during memory tasks [28]. In fact, retrieval deficits occur frequently as a consequence of poor initial encoding. 

While momentary confabulation is commonly seen in the WKS, spontaneous confabulation is relatively rare beyond the acute phase of the WE. The persistence of spontaneous confabulation rather suggests a concomitant damage to the frontal lobes, particularly the ventromedial and/or orbitofrontal regions [29]. There are conflicting theories about the underlying basis of spontaneous confabulation [29–31]. The content of confabulation depends on the confluence of different subcomponents of strategic retrieval [29]. The confabulating patients were likely to incorrectly recognise past autobiographical events or thoughts, and especially the unpleasant events, thus suggesting that also motivational factors contribute to confabulation [31].

Unlike non-WKS alcoholics, WKS patients still show, after protracted abstinence from alcohol significant impairment in visual and verbal short- and long-term memory, working memory, executive functions, general knowledge, and visuospatial abilities.

## 3. Cholinergic Dysfunction

Basal forebrain damage critically contributes to the diencephalic amnesia following thiamine deficiency in patients with WKS and experimental models [32, 33]. The basal forebrain region includes several groups of cholinergic neurons, medial septal area, diagonal vertical bands, and nucleus basalis of Meynert (NBM), that project to the hippocampus, amygdala, and cortex. The basal forebrain cholinergic complex projects directly to cortex and hippocampus and has minor connections with the thalamus [34]. Significant correlations were found between cortical and hippocampal acetylcholinesterase (AChE) activity and/or acetylcholine (ACh) release and measures of memory performance. The medial septum/diagonal band (MS/DB) complex located in the forebrain is composed of cholinergic and GABAergic neurons (which contain the calcium-binding proteins parvalbumin and calbindin) that project to the hippocampal formation [35]. This parallel system of basal forebrain GABAergic projections seems to complement the basal forebrain cholinergic complex.

However, the clinical and neuropathological data regarding the role of basal forebrain cholinergic cell loss in the pathophysiology of amnesia associated with alcohol-induced WKS are sometimes conflicting.

Arendt et al. reported a 25–45% reduction in cholinergic basal forebrain cells in the WKS patients, which was attributable largely (over 90%) to cholinergic cells of the MS/DB, although cell loss in the vertical limb of the DB and the NBM was also detected [36]. Current data suggest that a selective damage to the choline acetyltransferase (ChAT)-positive cells of the MS/DB, rather than a widespread cell death, contributes to the cell loss in the MS/DB region. A correlation has been found between the loss of cholinergic basal forebrain neurons and cognitive dysfunction, as assessed with the mini-mental state examination [32]. However, Cullen and coworkers reported that the cholinergic cell loss in the NBM is minor and failed to find a significant difference in the amount of cholinergic NBM cell loss in alcoholics with WE and alcoholics with the amnesic WKS [37]. Since these authors only included the NBM, it can be argued that the MS/DB cholinergic cell loss is mainly responsible for this specific type of cognitive dysfunction. In particular, activation of cholinergic forebrain projection neurons is thought to be important for encoding new stimuli in discrimination tasks [38]. The basal forebrain cholinergic neurons show differential vulnerability to neurotoxic events [39, 40]. 

The maintenance and survival of MS/DB cholinergic cells in the rodent (at variance with the NBM cholinergic neurons) are dependent on the neurotrophin nerve growth factor (NGF), which is predominantly synthesized by hippocampal GABAergic neurons that project to the MS [41–43]. 

The cholinergic fibers that project to the hippocampus retrogradely transport the NGF to the MS where it binds to the p75-NGF receptor [43]. In the rodent, unlike the MS/DB cholinergic neurons, the NBM cholinergic neurons that project to the amygdala do not express the p75-NGF receptor [44, 45] and are thus less sensitive to thiamine deficiency induced neurotoxicity [33]. Indeed, the presence of p75-NGF receptors on cholinergic neurons may make them sensitive to the neuronal death observed in the well-established animal models of WKS. Notably, also in degenerative neurological diseases such as Alzheimer's disease (AD) and Parkinson's disease, the degeneration process preferentially involves those cell populations that contain the p75-NGF receptor [46, 47]. 

## 4. Experimental Models of WKS 

In experimental models of thiamine deficiency, exposure to a thiamine-depleted diet for as little as 9 days in mice has been demonstrated to alter hippocampal neurogenesis at an early prepathological lesion stage, while the anatomy or physiology of the hippocampus is not substantially affected [48]. As thiamine deficiency progresses to 30 days, significant reduced levels of AChE in the cortex and hippocampus were observed [49]. This finding is associated with deficits in spatial memory performance on the Morris water maze task [49], as well as with impaired avoidance learning, as measured by passive-avoidance task [50, 51] and emotional behavior changes [51]. 

Cholinergic neurons are especially sensitive to the neurodegeneration caused by thiamine deficiency. ChAT-positive cell loss (about 25–30%) after thiamine deficiency well correlates with both, behavioral impairment and thalamic tissue loss. In contrast, the GABA neurons in the MS/DB are not significantly affected by thiamine deficiency [52]. Notably, also other neurological disorders characterized by degeneration of the cholinergic neurons have sparing of the GABAergic MS/DB neurons [32]. In particular, the corticopetal GABAergic system seems to be relatively preserved also in AD.

Although the exact mechanisms are unknown, cholinergic neurons death in the MS/DB after thiamine deficiency is not surprising. In fact, thiamine is critical for ACh synthesis [53] and ACh-synthesizing cells in the basal forebrain are especially liable to thiamine deficiency induced cell death [54]. Thiamine is a precursor for thiamine pyrophosphate, which serves as a cofactor for the production of several key enzymes, such as transketolase, alpha-ketoglutarate dehydrogenase, and especially pyruvate dehydrogenase, which plays an essential role in the conversion of pyruvate to acetyl-CoA during the process of glycolysis [53]. In addition, cholinergic cells appear to have a higher demand for energy production and are more sensitive to glucose deprivation [54]. The memory and other cognitive deficits observed during the later phases of thiamine deficiency TD are improved by administration of AChE inhibitors (AChEIs), the muscarinic agonist McN-A-343, or herbal compounds that increase ACh release [55]. After prolonged thiamine deficiency, there is also a loss of cholinergic neurons within the forebrain [48] and a loss of cholinergic fibers that innervate the hippocampus [50, 51] that could contribute to some of the cognitive/memory abnormalities seen in the late stages of thiamine deficiency. 

Pyrithiamine-induced thiamine deficiency (PTD) is another commonly employed animal model of diencephalic amnesia and has been used to study the neuroanatomical, molecular and behavioral consequences of TD [56–59]. The cerebral damage seen in rodents after PTD treatment shows many neuropathological and biochemical similarities to WE in humans and results in brain lesions, as well as learning and memory impairments, similar to those observed in humans with WKS. Indeed, dietary restriction alone typically produces lesions that are limited to brainstem nuclei but does not cause the pattern of diencephalic lesion observed in WKS [57, 59, 60], while the PTD model reproduces both the acute neurological symptoms of the acute WE, and the chronic neuropathological and cognitive deficits that are typical for alcohol-induced WKS [57, 59–61]. It involves the coadministration of pyrithiamine, an inhibitor of thiamine absorption and metabolism that also affects thiamine pyrophosphokinase and thiamine triphosphate [62], and reduces the complications and mortality rates that usually accompany dietary thiamine deficiency alone [61]. 

Alterations in basal forebrain cholinergic input to the hippocampus likely represent a key factor contributing to learning and memory impairment in the PTD model. Indeed, memory deficits during the WE stage of PTD are reversed via administration of AChEIs [55], indicative of cholinergic dysfunction. As above mentioned, a ~30% reduction in ChAT-positive cells in the MS/DB during the WKS stage has been demonstrated [52, 63, 64], which correlates with behavioral impairment on spatial tasks [52]. 

There is a close relationship between the cholinergic neuronal loss in the MS/DB, thalamic shrinkage, and behavioral dysfunction in the PTD rodent model of WKS. In this experimental model along with thalamic, cortical, and mammillary damage, there is also a loss of cholinergic neurons in the MS/DB [33, 63, 64]. Pyrithiamine treatment resulted in selective cell loss of ChAT-ir neurons that project from the MS/DB to the hippocampus. Indeed, *in vivo* microdialysis in PTD-treated rats revealed selective cholinergic hippocampal dysfunction [33, 65]. Moreover, administration of physostigmine by enhancing hippocampal ACh levels reversed the PTD-induced behavioral impairment [66]. This finding further suggests that there is a functional impairment of the septohippocampal pathway in PTD-treated rats. 

Anzalone et al. [67] found a blunted efflux during behavioral testing and significant reductions in cholinergic fiber densities in the hippocampus, medal frontal cortex, and retrosplenial cortex, but cholinergic fiber density correlated with Ach efflux only in the hippocampus. In particular, the diencephalic-hippocampal interaction appears thus to be critical for successful episodic and spatial learning/memory. However, not only the hippocampus but also the limbic (prefrontal, frontal, and retrosplenial) cortices are functionally affected by PTD treatment. Accordingly, fluorodeoxyglucose-positron emission tomography shows in WKS patients hypometabolism in diencephalic-limbic grey matter [68]. It should be noted that some structures related to memory processes are not affected; cholinergic activation in the amygdala and striatum is normal when rats are tested on hippocampal-dependent tasks (i.e., spontaneous alternation task) [3, 33].

A combined neuropathology to diencephalic and basal forebrain systems may result in the behavioral impairment observed in the PTD model and in patients with WKS.

In fact, lesion of either the medial thalamus [69, 70] or the of the cholinergic septohippocampal system [71, 72] alone can produce behavioral impairment. However, there is a probably synergistic interaction between neuropathology in the PDT model. In fact, the severity of behavioral impairments seen in the PTD model tends to be greater and broader. While damage to the cholinergic septohippocampal pathway and thalamic tissue loss predict behavioral dysfunction, the GABAergic interneurons and projection neurons are spared. 

Thus, therapeutic approaches should address both of these neuropathological outcomes that occur in diencephalic amnesia associated with alcoholism and thiamine deficiency. Cholinergic septohippocampal dysfunction significantly seems to contribute to the amnesic disturbances that occur after thiamine deficiency. It is still unknown whether there is a common causal neurotoxic mechanism across the MS/DB and thalamus or if the neuropathological abnormalities occur independently. Anyway, both thalamic tissue damage and cholinergic cell loss in the MS/DB are key features of diencephalic amnesia associated with thiamine deficiency and occur in parallel. Although it is still unclear whether the cholinergic neurons in the MS/DB are selectively sensitive to neurodegenerative processes induced by thiamine deficiency, the strong correlation between thalamic mass and cholinergic neurons in the MS/DB is highly suggestive of a common pathogenic link. 

## 5. Glutamate Excitotoxicity

Thiamine deficiency has been proposed to lead to mitochondrial dysfunction that determines impaired cellular metabolism, glutamate excitotoxicity, and oxidative stress in the thalamus, mammillary bodies, and other diencephalic structures [53, 73]. Glutamate plays a significant role in the development of thiamine deficiency lesions. In fact, glutamate excitotoxicity is the primary mechanism of cell death in the PTD model associated with diencephalic amnesia [74, 75]. The neurocytopathological changes induced by PTD are identical to those that have been described in glutamate-induced excitotoxic lesions [76]. Excitotoxic and possible apoptotic mechanisms may mediate neuronal degeneration in the PTD rat model of WE and WKS. The absence of any increase in glutamate levels in a nonvulnerable region such as the frontoparietal cortex further supports the theory that a glutamate-mediated excitotoxic mechanism may be responsible for the vulnerability in thiamine deficiency [74]. Early microglial activation and increased abnormal metabolism (increased production) of free radicals have been reported in rats after thiamine deficiency. These findings strongly suggest that the role of oxidative stress in the brain damage is associated with thiamine deficiency [77]. Reduction in thiamine and thiamine-dependent processes may lead to neurodegeneration and determines oxidative stress. Elevated levels of the oxidative stress indicators hemeoxygenase-1 and intracellular cell adhesion molecule-1 have been demonstrated in the brain of rats after thiamine deficiency [78]. The neuroanatomical damage seen after PTD treatment is most likely due to glutamate excitotoxicity and/or ischemia [53, 74]. It has been well documented that glutamate excitotoxicity is responsible for thalamic lesions in the PTD model of WKS [75, 76] and cholinergic neurons are particularly sensitive to glutamate excitotoxicity. In particular, the differential susceptibility of cholinergic populations to oxidative stress depends on the ratio of acetyl-CoA energy producing and ACh synthesizing capacities [79]. The extent of thalamic damage correlates strongly with the number of MS/DB ChAT-ir cells, as well as with behavioral impairment on the spontaneous alternation task. This type of behavioral dysfunction in PTD rats is correlated to reduced hippocampal ACh release [65] and may lead to a restored behavioral performance by increasing hippocampal ACh levels [66]. 

## 6. Neuroanatomical Findings

Numerous studies have revealed some significant neuropathological differences between WKS and non-WKS alcoholics. It has been demonstrated [7, 80] that thiamine deficiency is the foremost feature causing brain damage and structural changes in alcoholics. While non-WKS alcoholics display some degree of structural abnormalities in the same regions as WKS alcoholics, these are in the vast majority of the cases less severe. 

In WKS patients, independent from the etiology of thiamine deficiency (different diseases or conditions that result in restrict eating and/or inadequate vitamin absorption), a prominent neuropathological consequence is lesions of the anterior and midline thalamic nuclei as well as the mammillary bodies [81, 82], and this extent of pathology is not evident in alcoholics without WKS. 

Sulcal and Sylvian fissure widths are equivalent in WKS and non-WKS alcoholics [83]. However, WKS patients have wider third ventricles, larger lateral ventricles, and wider interhemispheric fissures than non-WKS chronic alcoholics [84]. 

There is a continuum of pathology between the two ADD and WKS. In fact, a grading pattern of volume deficits can be observed from mild in non-WKS alcoholics to moderate and severe in WKS, in pons, mammillary bodies, hippocampus, thalamus, cerebellum, and the frontal cortex [84]. Among the cerebral regions, the frontal lobes were found to be most vulnerable to alcohol-related brain damage [15, 18, 85, 86]. There is a modest loss of cholinergic innervation of the frontal cortex. However, during behavioural tests in the PDT model a lack of cholinergic activation in the frontal cortex was observed, thus suggesting that frontal cortex is a “functionally lesioned” structure.

In the PTD animal model, during the early WE stage, anatomical damage is localized to the gelatinosus and anteroventral ventrolateral thalamic nuclei [76]. At the later WKS stage, cell death extends into the posterior thalamus (ventroposterolateral, ventroposteromedial, and ventrolateral nuclei) [76, 87]. 

As in the animal models the encephalopathy progresses to the WKS stage, the pathological damage further expands and involves the medial mammillary bodies and numerous midline intralaminar thalamic nuclei [57, 88]. The amnesic syndrome in WKS is due to the damage in the diencephalic-hippocampal circuitry including thalamic nuclei and mammillary bodies. In particular, the neuronal loss in the anterior thalamic nuclei (ATN) was found to be critical to the production of amnesia in alcoholic-WKS patients [89]. There is also a degeneration of functionally important limbic system fiber tracts (i.e., fimbria/fornix and mammillothalamic tract) [75, 90], while a severe hippocampal damage is rare despite the evidence of functional impairment [3, 65]. The ATN are of crucial importance for learning and memory processes, and isolated damage to this region produces a persistent amnestic syndrome. Since dense connections exist between the ATN and the hippocampus, damage to the ATN can impair hippocampal functioning [66]. 

Therefore, cholinergic dysfunction associated with diencephalic amnesia is likely the consequence of an impaired cortical plasticity during cognitive processing rather than the result of anatomical changes after deafferentation. 

## 7. Conclusion

To the best of our knowledge, this is the first comprehensive review article which covers the most relevant neurochemical, neuroanatomical, and neuropsychological findings that have been reported after thiamine deficiency.

WKS occurs for any conditions that may cause malnutrition and thiamin deficiency. It is most commonly observed in people with many years of chronic alcoholism. In fact, chronic alcoholics are often in a malnourished state because alcohol interferes with the absorption of nutrients from the digestive system and replaces foods needed for essential nutrients. Thiamin is required for energy production and proper functioning of neurons. Thiamin deficiency can lead to damage or death of neurons. Insufficient levels of thiamin damage more severely some selectively vulnerable regions of the brain, in particular the thalamus (the major relay station of the brain that serves many important functions) and the mammillary bodies, which are part of the hypothalamus, located just below the thalamus. The mammillary bodies receive many neural connections from the hippocampus, which appears to be the primary part of the brain involved in the formation of memories, and mammillary bodies neurons projects to the thalamus, which in turn makes connections with the cerebral cortex, where long-term memories are stored. These anatomo-functional correlations may explain why lesions involving the thalamus and the mammillary bodies can lead to anterograde amnesia. Memories formed in the hippocampus cannot be stored since connections within the cortex are disrupted. Particularly the ATN is important for learning and memory, and damage to this region produces a persistent amnestic syndrome.

PTD was used to produce a rodent model of WKS that results in acute neurological disturbances, thalamic lesions, and learning and memory impairments. ACh is a key neurotransmitter, and *in vivo* measures of ACh in the hippocampus are correlated with learning and memory performance. The loss of diencephalic and in particular the loss of cholinergic neurons in the MS/DB can contribute to the neuropsychological impairments and blunted behaviorally evoked hippocampal ACh efflux in the PTD model [91]. However, ATN lesions did not affect basal concentrations of ACh in the hippocampus [91]. Therefore, the specific neurochemical substrate underlying the amnesia in patients with WKS is still poorly defined. A transcranial magnetic stimulation (TMS) technique, the short latency afferent inhibition (SAI), may give direct information about the function of some cholinergic pathways in the human motor cortex [91]. Nardone et al. [92] provided physiological evidence of cholinergic involvement in WKS. However, this putative marker of central cholinergic activity did not significantly correlate with the memory deficit in the examined patients. These findings suggest that the cholinergic dysfunction alone is not sufficient to cause a persisting amnesic syndrome in WKS. Multiple neurochemical abnormalities may thus underlie this memory loss in WKS. In particular, the noradrenergic and serotonergic systems have been implicated in the pathophysiological mechanisms of the WKS [92, 93], and the noradrenergic locus coeruleus [94] and the brainstem serotoninergic nuclei [95] have been examined in detailed neuropathological studies. A better understanding of mechanisms involved in pathophysiology of thiamine deficiency may also enable early detection and intervention in patients with WKS. 
